# Conjunctival Ultraviolet Autofluorescence as a Measure of Riboflavin and Ultraviolet and Accelerated Cross-Linking Exposure in Keratoconic Patients

**DOI:** 10.3390/jcm9092693

**Published:** 2020-08-20

**Authors:** Arleta Waszczykowska, Krzysztof Bartosiewicz, Michał Podgórski, Ewa Zmysłowska-Polakowska, Piotr Jurowski

**Affiliations:** 1 Department of Ophthalmology and Vision Rehabilitation, 2nd Chair of Eye Diseases, Medical University of Lodz, Żeromskiego 113, 90-549 Łódź, Poland; krzysztof.bartosiewicz24@gmail.com (K.B.); piotr.jurowski@umed.lodz.pl (P.J.); 2 Department of Diagnostic Imaging, Polish Mother’s Memorial Hospital Research Institute, Rzgowska 281/289, 93-338 Lodz, Poland; chilam@tlen.pl; 3 Department of Endodontics, Medical University of Lodz, Pomorska 251, 92-216 Łódź, Poland; ewa.zmyslowska-polakowska@umed.lodz.pl

**Keywords:** conjunctival ultraviolet autofluorescence, CUVAF, keratoconus, corneal cross-linking, aCXL, ultraviolet A

## Abstract

Purpose: The study was performed to analyze the prevalence of the conjunctival ultraviolet autofluorescence (CUVAF) area in keratoconic eyes and changes caused by UVA-irradiation as a component of accelerated corneal cross-linking (aCXL). Methods: The study group involved 20 keratoconic patients subjected to aCXL surgery in one eye. The comparative group consisted of 111 age- and sex-matched patients with healthy corneas. The images of the anterior segment in both patient groups were taken using a Coroneo camera. In the study group the photos were taken before and immediately after the surgery, and 7 and 30 days following the procedure. Results: Nasal and temporal autofluorescence area (AN+T) were significantly smaller in a keratoconic patients group compared to control group (*p* = 0.0001). Patients with the third stage of keratoconus had significantly higher AN+T (*p* = 0.0277) compared with individuals with lower stage keratoconus. No statistically significant CUVAF changes were observed after the aCXL procedure. In keratoconic patients with primary CUVAF undergoing aCXL, a temporary fast enlargement of the autofluorescence area was observed. Conclusions: The eyes undergoing the aCXL procedure showed no difference in the size of the CUVAF area but such patients should be in strict follow-up in order to reveal UV-related ocular surface diseases.

## 1. Introduction

Conjunctival ultraviolet autofluorescence (CUVAF) photography is reported to be a novel biomarker of the preclinical ocular surface sunlight-induced UV damage [1,2]. Excessive ocular ultraviolet radiation exposure is the pathogenic factor for numerous diseases including pterygium, ocular surface squamous neoplasia and acute photokeratitis [3]. The technique derives from Wood’s lamp which is often used in dermatological investigations. Wood’s light is commonly used to directly evaluate abnormally accumulated endogenous chromophores and pigmentation disorders, exogenous chromophores or porphyrin compounds that fluoresce in the visible spectrum during UV exposure [4]. A similar phenomenon occurs on the conjunctiva. Conjunctival fluorescence can be recorded with a camera system and analyzed digitally to determine the extent of ocular sun damage [1]. The technique has been shown to have excellent reliability, with intra- and interobserver concordance correlation coefficients exceeding 0.900 [5]. The validity of the technique as a biomarker of ocular sun exposure and outdoor time has been demonstrated by correlation with self-reported outdoor time measured by questionnaire, with p_trend_ across categories of outdoor time <0.001 [5,6].

Several investigations confirmed the clinical and epidemiological benefits of measuring CUVAF area, although the biological link between the UV-related damage and CUVAF is still unknown [2,7,8]. The measurement of ocular sunlight exposure has a key use for epidemiological research and preventive strategies for ophthalmic diseases associated with insufficient or excessive ultraviolet radiation (UVR) [5]. A positive correlation between the long time outdoors, increased size of CUVAF area and risk of ophthalmohelioses has been proven [3]. It is also believed that the progression of myopia is related to less time outdoors and thus the smaller area of CUVAF [8].

Corneal cross-linking (CXL) is a procedure enhancing the biomechanical properties of the cornea in progressive keratoconus (KC). In this method, the cornea is first instilled with riboflavin and subsequently UVA-irradiated with a total energy dose (fluence) of 5.4 J/cm^2^ [9]. Standard CXL methodology involves removal of corneal epithelium (Epithelium-Off) to improve penetration of riboflavin (vitamin B_2_), as epithelium provides a natural diffusion barrier for this compound. To avoid UVA-induced damage to the corneal endothelium, the parameters for CXL are set to providing effective treatment in the anterior 300 μm of the corneal stroma. Nevertheless, no additional interventions to protect the ocular surface are undertaken [10]. Numerous modifications of the standard CXL technique were implemented so far, some of which aimed at the protection of the corneal epithelium (transepithelial or Epithelium-On method), while others, at the reduction of the procedure duration (accelerated CXL method, aCXL) [11].

In the present study we raised two clinical problems comprising the prevalence and degree of changes in the CUVAF area in keratoconic eyes versus the healthy population, and the assessment of aCXL safety according to comparison of the conjunctival fluorescence before and after the aCXL procedure in patients with keratoconus vs. untreated eyes.

## 2. Methods

### 2.1. Participants

The study group involved 20 keratoconic patients (mean age 33.3 ± 11.0 years; 18 men, 2 women) with documented progression of keratoconus within the last 12 months (an increase of astigmatism or myopia by 1 D or an increase of K_max_ by 1 D). Each patient underwent the aCXL in one eye. The aCXL procedure was based on irradiation with light at the power of 6 mW/cm^2^ and 365 nm wavelength (UVA) (Opto XLink-corneal crosslinking system, Opto Electronica S/A, Sao Paulo, Brazil) for 15 min with simultaneous application of 0.1% riboflavin in 20% Dextran 500 solution (Opto Ribolink, Opto Global Pty Ltd., Adelaide, Australia). Moreover, balanced salt solution (BSS) was applied every 6 min to moisten the cornea. The details of the mode of procedure were described previously [11].

The stage of keratoconus was determined for both eyes according to Amsler–Krumeich’s classification by using Placido’s disk videokeratography (Keratograph 4, Oculus Inc., Wetzlar, Germany). Baseline and intraoperative corneal pachymetry (handheld device PacScan 300AP, Sonomed Inc., Lake Success, NY, USA) was performed in the keratoconus apex determined by videokeratography. Inclusion criteria for this study were—corneal thickness of at least 400 μm at the keratoconus apex, 2nd or 3rd stage of keratoconus according to the above-mentioned classification and the withdrawal of wearing rigid contact lenses for 4 weeks before the procedure. Exclusion criteria included—corneal thickness <400 μm, dry eye syndrome, history of viral corneal inflammation, past ophthalmologic surgeries, cornea guttata, corneal scars, pregnancy, autoimmune diseases and chronic corticosteroid therapy. All the patients qualified for the procedure were adults and signed informed consent for the surgery.

The comparative group consisted of 111 age and sex matched patients with healthy corneas (mean age 31.7 ± 12.5 years, *p* = 0.4758; 100 men, 11 woman, *p* = 0.9901), without any ocular, genetic, or systemic diseases and without previous ocular surgery. Exclusion criteria included also sunbed use and intended sun exposure in the previous 6 months.

The study was conducted in line with the ethical principles specified in the Declaration of Helsinki of 1964. Informed consent was obtained from all participants after explanation of the nature and possible consequences of the study. Approval of the local Bioethics Committee was obtained (No. RNN/508/11/KB). All baseline studies and aCXL treatments in the KC patients group were conducted in January, the control group studies were conducted between February and March 2016.

### 2.2. Conjunctival Ultraviolet Autofluorescence

Conjunctival ultraviolet autofluorescence photographs were taken using the camera with a specially-adapted electronic flash and filter system developed by Coroneo and colleagues [1]. This consisted of a Nikon D100 (Nikon, Melville, New York, NY, USA) digital camera and 105 mm f/2.8 Micro Nikkor (Nikon, Melville) lens fitted with infrared and UV barrier filters (B&W 489 and B&W420 and rotating polarizer; transmittance range 300–400 nm, peak 365 nm) as an excitation source, so primarily the UV autofluorescence was captured by the camera sensor. The flash unit was a Metz 36C-2 (Zirndorf, Germany–guide number 36 (m)/ISO 100/21°) overlaid with Wratten 2E and 18A UV (Kodak, Rochester, NY, USA) transmission filters. A white LED light fixed to the chinrest was used to assist in positioning and focusing, yet was turned off prior to image acquisition. Photographs were taken in a dark room so only ultraviolet fluorescence was recorded by the camera. Images were saved in the RGB format at the D100 JPEG Fine (1:4 compression) and high resolution settings.

**Figure 1:**
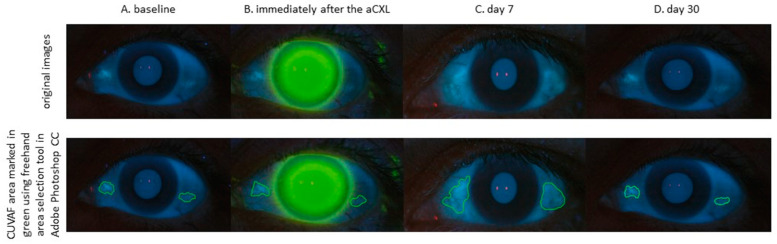
Panel of 8 conjunctival ultraviolet autofluorescence digital images of the left eye of a patient aged 28, diagnosed with keratoconus in the 3rd stage according to the Amsler classification. Photos taken during the study visits—baseline, immediately after the aCXL procedure, day 7, and day 30. Photos presented in the original and after processing in Adobe Photoshop CC with manual marking of the CUVAF area in green. Abbreviations: aCXL, accelerated corneal cross-linking; CUVAF, conjunctival ultraviolet autofluorescence.

CUVAF images were obtained for temporal and nasal regions of each eye for 111 control subjects. In keratoconic patients group, the photographs of both eyes were taken before and immediately after the surgery and on 7 and 30 days after the procedure (Figure 1).

Analysis was performed independently by two experienced ophthalmologists (AW and KB) using Adobe Photoshop CC (Adobe Photoshop CC Version 20.0.6, Adobe Systems Inc., San Jose, CA, USA) and the results were averaged (Figure 1). Four photographs were analyzed for each participant (left and right eyes, nasal and temporal conjunctiva). Each area of the nasal (AN) and temporal (AT) regions was measured twice and the average obtained (AN+T). In addition, we performed measurements in the nasal (ApN) and temporal perilimbal conjunctival area (ApT) 2.0 mm wide. Subsequently, total ultraviolet autofluorescence in mm^2^ was calculated by adding the average nasal and temporal areas for each eye.

### 2.3. Statistical Analysis

The Statistica 13.0 Software (Statsoft, Cracow, Poland) was used for calculations. Data are presented as a mean and standard deviation (SD) and *p* < 0.05 was considered significant. Based on power analysis, the minimum study sample should be 14 individuals. Normality of data distribution was checked with the Shapiro–Wilk test and due to skewed distribution nonparametric test were used. In a group with keratoconus for comparison of values between eyes before the therapy, the Wilcoxon signed-rank test was applied. To assess the effect of treatment, consecutive measurements in threated eyes were compared with the Friedman’s test with dedicated post hoc tests. To compare results of CUVAF between two groups (including both eyes in a study group and different time from the procedure), the Kruskal–Willis test was applied with dedicated post hoc tests.

## 3. Results

In the study group, the eyes treated with aCXL (six right and 14 left) presented in the 2nd (17 patients) and 3rd (three patients) stage of keratoconus. In the contralateral eye, there was no keratoconus (four patients), or 1st stage (seven patients), or 2nd stage (six patients), or 3rd stage (three patients) of keratoconus. Eyes with the 3rd stage of keratoconus had significantly larger AN+T compared to eyes with lower stage of KC (*p* = 0.0277), while ApN+T did not differ significantly (*p* = 0.0842).

In Table 1 there are values of A and Ap parameters compared between the treated eye and the control group while in Table 2 between treated and untreated eyes of the same patient.

According to post hoc analysis, values of AN+T in eyes undergoing aCXL procedure were significantly lower in day 0 (both before and after procedure) than in the control group. While values of ApN+T in eyes undergoing the aCXL procedure were significantly lower in all time points when compared with the control group. These differences were significant due to changes in the nasal side, because values for AN and ApN in eyes undergoing aCXL procedure were significantly lower in all time points when compared with the control group. For the temporal side the opposite was observed, but only values of AT in eyes undergoing aCXL procedure were significantly higher 30 days after the procedure when compared with the control group.

**Figure 2:**
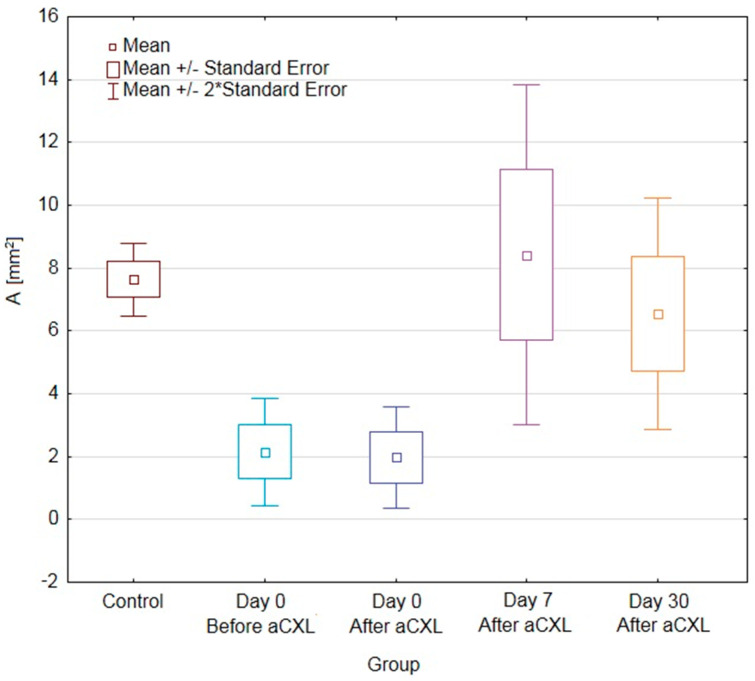
Box-and-whiskers plot of conjunctival ultraviolet autofluorescence (CUVAF) nasal and temporal area (AN+T) measurements in keratoconical eyes undergoing accelerated corneal cross-linking (aCXL) procedure and untreated keratoconical eyes as well as the eyes of control group patients. Abbreviations: CUVAF, conjunctival ultraviolet autofluorescence; AN+T, nasal and temporal conjunctival autofluorescence area; aCXL, accelerated corneal cross-linking.

**Figure 3:**
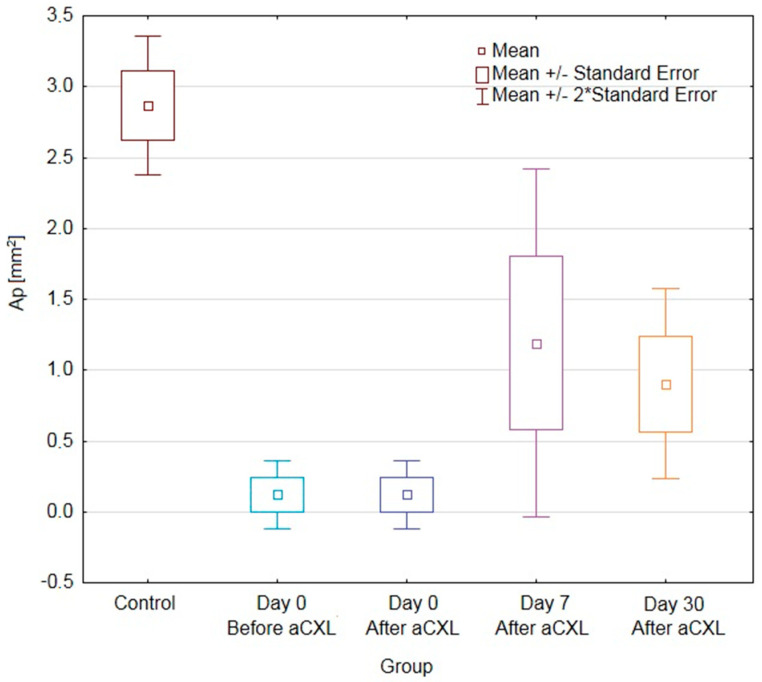
Box-and-whiskers plot of conjunctival ultraviolet autofluorescence (CUVAF) nasal and temporal perilimbal area (ApN+T) measurements in keratoconical eyes undergoing accelerated corneal cross-linking (aCXL) procedure and untreated keratoconical eyes as well as the eyes of control group patients. Abbreviations: CUVAF, conjunctival ultraviolet autofluorescence; ApN+T, nasal and temporal perilimbal conjunctival autofluorescence area; aCXL, accelerated corneal cross-linking.

When comparing values only for the treated eye for both nasal and temporal side there was a trend towards increasing values of autofluorescence from day 0 before procedure, through day 0 after the procedure till day 7, with decreasing values 30 days after the procedure. However, only differences for AN+T and ApN+T were significant (*p* = 0.0003 and *p* = 0.0025, respectively) and it was due to significantly higher values of AN+T and ApN+T in day 7 than in day 0 after the procedure (Figure 2 and Figure 3).

The CUVAF area in the untreated eye did not differ statistically at any stage of the study. In comparison between the treated and fellow eye, AN+T and ApN+T in eyes undergoing the aCXL procedure were significantly lower in day 0 (both before and after procedure) than in the opposite eye (Table 2). On the other hand, the day 7 increase in AN+T and ApN+T parameters in eyes undergoing aCXL made them significantly higher than in the fellow eye. In day 30 the differences were not significant. The above was also true for AN but not for other parameters.

## 4. Discussion

The present study was performed to analyze the prevalence of the CUVAF area in keratoconic eyes, and to determine the changes caused by the UVA-irradiation as a component of accelerated corneal cross-linking, as well as to compare it with a control group of patients with healthy corneas.

To the best of our knowledge, there are no reports concerning the CUVAF area in keratoconic patients, although a few studies have previously indicated a positive correlation between the exposure to ultraviolet light and keratoconus as an environmental factor in the etiology of the disease [12].

Our investigation had five important results. Firstly, keratoconic patients presented smaller CUVAF areas compared to the control group. Secondly, the location of CUVAF differed significantly in both studied groups. Thirdly, we observed a positive correlation between the size of CUVAF area and the grade of the keratoconus advancement. Fourthly, we observed an increased tendency to transiently enlarge the CUVAF areas in patients undergoing aCXL surgery, in whom we observed the presence of CUVAF in the baseline study. Finally, in a 30-day observation, the size of the CUVAF area remains unchanged after aCXL surgery, which proves the safety of the procedure.

However, our study has several limitations. Unfortunately, we did not collect data in the patients’ history regarding the sun protection used and the time spent outside. A likely explanation for the smaller size of CUVAV in keratoconus patients may be the widespread use by KC patients of corrective glasses and contact lenses, which usually contain a UV filter.

The cornea is particularly susceptible to UV due to its natural transparency and shape. The well-known Coroneo effect (the ability to refract light rays through the cornea and focus them 20-fold stronger in the nasal conjunctival region than temporal) may explain the higher incidence of CUVAF in the nasal region in a healthy population [13,14]. These observations are consistent with our findings, as CUVAF was more frequent in the nasal part of the conjunctiva in the control group with healthy corneas. Previous studies have shown that differences in corneal topography can account for the clinical observation of individual variation in the degree of limbal light focusing [15]. Corneas with steeper radii focus light more anteriorly and more intensely [15,16]. As the keratoconus increases, the focus of the lateral rays in the nasal area expands to the outside of limbus [16]. This is consistent with our results. We have observed significantly larger CUVAF area in the AN sector in patients with the 3^st^ stage of keratoconus while the ApN sector CUVAF did not differ between patients with a lower disease severity. We are aware that the corneal refractive power also has an impact on the radiation focus. It is increased with thinner central corneal thickness, deeper anterior chamber, shorter axial length, and myopic refractive error [17], the values of which we have not studied in our patients. All these factors may also affect the different location of light rays depending on the angle of incidence compared to the control group. A positive correlation between the CUVAF area size and the grade of the keratoconus advancement may be related to the greater collagen lesion [18] presence and thus greater susceptibility to UV damage in proportion to the stage of the progression of the disorder [19]. It is also possible that, as in fluorescence spectroscopy of human skin, the fluorescence excitation spectrum is diverse due to changes in the molecular environment of the fluorophores [20]. It is known that upregulated levels of metalloproteinases and pro-inflammatory cytokines correlated with the severity of the keratoconus [19,21]. It also seems that UV radiation itself activates a number of pro-inflammatory molecules, such as various interleukins, cytokines and matrix metalloproteinases, which are responsible for cell injury [22,23].

Previous research efforts have focused mainly on the impact of UVB radiation on human health, which is connected to chronic skin inflammation and melanoma [24]. UVA radiation carries lower energy and, therefore, is considered to have a less damaging effect. However, recent reports show that UVA is the main factor causing changes to the stromal extracellular matrix and photoageing of the cornea [25]. The UVA dose adapted in CXL procedure is 60 W/m^2^ in determining the exact dose of radiation affecting the cornea and conjunctiva [11]. The value of UV index for Poland for natural solar radiation according to the Central Geophysical Observatory Belsk for January, February and March varies between 1–2, which corresponds to a UVR dose below 0.7 W/m^2^ [26]. In our study, UVA radiation during CXL treatment was the only common factor of the UV exposure, as the treated patients limited their time spent outdoor in the postoperative period, thereby limiting additional damage related to sunlight. The CUVAF area in the fellow eye did not differ statistically at any stage of the study, which allows us to exclude the influence of environmental factors on the assessment of CUVAF changes after the aCXL procedure.

Analyzing our results of CUVAF measurements in the days following the aCXL procedure, we observed that in the eyes in which initially no UV-related conjunctival lesions were found, the CUVAF area did not appear after the procedure or increased minimally during the observation period. On the other hand, in the eyes showing the presence of CUVAF in the baseline examination, the tendency of their enlargement or the formation of new areas in the first days after the procedure were determined. Unfortunately, we did not manage to demonstrate statistical significance in our observations probably due to the small size of the examined group. These facts may prove an individual susceptibility to UV damage and thus suggest that in people susceptible to UV damage, even a safe dose of radiation may cause an exacerbation of CUVAF areas [12].

During the first 7 days after the aCXL procedure in our keratoconic patients, the enlargement of conjunctival lesions was observed (*p* = 0.0006), which lessened during long-term observation and returned to the baseline after 30 days. CUVAF may indicate short-term UV damage similar to a skin tan [21]. The dermatological reports reveal that UV-induced changes in the skin tissue structure may be reversible if excessive sun exposure is limited, whereas iterative exposure to UV light promotes accumulative defects [22]. The observed transient growth of CUVAF area after aCXL surgery can be facilitated by remodeling of collagen and changes in the intracellular content of proteins including cytokines and matrix metalloproteinases [21,27].

It is unclear from current literature if CUVAF is indicative of accumulative UV damage [2]. The study results on the effects of seasonal variability on the CUVAF area are limited and contradictory. So far, the association between air temperature and thus UV intensity to the amount of CUVAF has not been studied [2]. Our observations were carried out during the winter months in order to limit as much as possible the influence of the temperature on the intensity of UV radiation in the control group. Infrared thermocamera measurements of the corneal surface during aCXL treatments showed an average temperature of 31.5 °C during the entire procedure, below the threshold of thermal collagen damage [28]. However, adverse effects in the limbal epithelial cell compartment after CXL surgery have been reported. It has been shown that UVA treatment promoted the upregulation of genes connected to apoptosis [29], while signs of oxidative damage were found in basal limbal epithelial cells positive for the stem cell marker P63α, thus suggesting a link of UVA to an impairment of the limbal stem cell population [30].

Our observations indicate that the exacerbation of conjunctival staining is a self-prompting process in which the primary CUVAF is more susceptible to UV-related damages de novo. This further suggests that the CUVAF is a very sensitive indicator of subacute deterioration associated with UV radiation. Hence, the patients undergoing cross-linking should be in strict follow-up in order to reveal UV-related ocular surface diseases, such as pterygium or neoplasia.

### Limitations

The influence of corneal ectasia presence on the promotion of changes in UVA radiation-induced CUVAF is arguable. Our investigation excluded UVA-irradiation changes of healthy corneas and therefore we suggest this relates only to keratoconic patients. Moreover, the influence of riboflavin instillation during the CXL is also uncertain concerning the effect on conjunctival epithelium, as the drug has not been administered to eyes without procedure.

Unfortunately, we did not have a history of the applied protective correction against ultraviolet radiation and time spent outdoors in any of our patient groups.

## Figures and Tables

**Table 1:** Comparison of autofluorescence parameters between control group and eyes treated for keratoconus. Parameters presented in mm^2^ as a mean and (SD).

Parameter	Control	Study Group-Eye with aCXL				*p* for All Subgroups
		Day 0 before aCXL	Day 0 after aCXL	7 Days after aCXL	30 Days after aCXL	
AN	7.16 (4.82) *	0.89 (1.61) *	0.94 (1.64) *	3.94 (5.91) *	2.11 (3.64) *	0.0001
ApN	2.64 (1.94) *	0.00 (0.00) *	0.00 (0.00) *	0.62 (1.55) *	0.18 (0.60) *	0.0001
AT	0.48 (1.19) *	1.26 (3.04)	1.04 (3.02)	4.48 (7.41)	4.43 (5.59) *	0.0001
ApT	0.23 (0.67)	0.12 (0.54)	0.13 (0.56)	0.57 (1.39)	0.72 (1.22)	0.1092
AN+T	7.64 (5.07) *	2.15 (3.79) *	1.97 (3.55) *	8.42 (12.11)	6.54 (8.22)	0.0001
ApN+T	2.87 (2.13) *	0.12 (0.54) *	0.12 (0.54) *	1.19 (2.74) *	0.90 (1.50) *	0.0001

Abbreviations: A, autofluorescence area; AN, nasal conjunctival autofluorescence area; ApN, perilimbal nasal conjunctival autofluorescence area; AT temporal conjunctival autofluorescence area, aCXL, accelerated cross-linking; *—indicates significant differences according to the post hoc test.

**Table 2:** Comparison of autofluorescence parameters between eye treated for keratoconus and the fellow, untreated eye. Parameters presented in mm^2^ as a mean and (SD).

Study Group-Eye with AND without aCXL									
Parameter	Day 0 before aCXL		Day 0 after aCXL		7 Days after aCXL		30 Days after aCXL		*p* for All Subgroups
	T Eye	Ut Eye	T Eye	UT Eye	T Eye	UT Eye	T Eye	UT Eye	
AN	0.89 (1.61) *	2.10 (2.36) *	0.94 (1.64) *	2.05 (2.34) *	3.94 (5.91) *	2.06 (2.38) *	2.11 (3.64)	2.01 (2.27)	0.0141
ApN	0.00 (0.00)	0.15 (0.38)	0.00 (0.00)	0.15 (0.37)	0.62 (1.55)	0.14 (0.39)	0.18 (0.60)	0.14 (0.37)	0.0941
AT	1.26 (3.04)	2.88 (4.07)	1.04 (3.02)	2.91 (4.15)	4.48 (7.41)	2.87 (4.00)	4.43 (5.59)	2.90 (4.04)	0.0568
ApT	0.12 (0.54)	0.54 (1.02)	0.13 (0.56)	0.55 (1.07)	0.57 (1.39)	0.54 (1.04)	0.72 (1.22)	0.55 (1.06)	0.1092
AN+T	2.15 (3.79) *	4.98 (5.80) *	1.97 (3.55) *	4.96 (5.78) *	8.42 (12.11) *	4.93 (5.74) *	6.54 (8.22)	4.91 (5.78)	0.0193
ApN+T	0.12 (0.54) *	0.69 (1.30) *	0.12 (0.54) *	0.70 (1.28) *	1.19 (2.74) *	0.68 (1.25) *	0.90 (1.50)	0.69 (1.29)	0.0286

Abbreviations: A, autofluorescence area; AN, nasal conjunctival autofluorescence area; ApN, perilimbal nasal conjunctival autofluorescence area; AT temporal conjunctival autofluorescence area, aCXL, accelerated cross-linking; T eye—treated eye; UT eye—untreated eye, *—indicates significant differences according to the post-hoc test.
